# Involvement of miR-619-5p in resistance to cisplatin by regulating ATXN3 in oral squamous cell carcinoma

**DOI:** 10.7150/ijbs.54014

**Published:** 2021-01-01

**Authors:** An Song, Yuanyuan Wu, Weiming Chu, Xueming Yang, Zaiou Zhu, Enshi Yan, Wei Zhang, Junbo Zhou, Xu Ding, Jie Liu, Hongxia Zhu, Jinhai Ye, Yunong Wu, Yang Zheng, Xiaomeng Song

**Affiliations:** 1 Jiangsu Key Laboratory of Oral Diseases, Nanjing Medical University, Nanjing, Jiangsu, People's Republic of China.; 2 Department of Oral and Maxillofacial Surgery, Affiliated Hospital of Stomatology, Nanjing Medical University, Nanjing, Jiangsu, People's Republic of China.; 3 Clinical Medical College, Yangzhou University, Yangzhou, Jiangsu, People's Republic of China.; 4 Department of Stomatology, the Affiliated People's Hospital of Jiangsu University, Zhenjiang 21200, Jiangsu Province, China.; 5 Department of Anesthesiology, Affiliated Hospital of Stomatology, Nanjing Medical University, Nanjing, Jiangsu, People's Republic of China.; 6 Department of Stomatology, Nanjing Integrated Traditional Chinese and Western Medicine Hospital, Nanjing, Jiangsu, People's Republic of China.

**Keywords:** OSCC, miR-619-5p, cisplatin-resistance, ATXN3

## Abstract

MicroRNAs are major post-transcriptional regulators responsible for the development of human cancers, including OSCC. The specific role of miR-619-5p in OSCC, however, is rarely reported. Cisplatin is one of the mostly applied chemotherapy drugs of OSCC. Nevertheless, drug resistance of cisplatin following the initial chemotherapy largely restricts its clinical benefits, and the mechanism of cisplatin resistance is unclear. This study intends to explore the biological function of miR-619-5p in the development of cisplatin resistance in OSCC cell lines and a xenograft model, as well as the potential molecular mechanism. Our results showed that miR-619-5p was down-regulated in OSCC samples and cisplatin-resistant OSCC cells. Ectopically expressed miR-619-5p inhibited proliferative, migratory and invasive abilities of OSCC cisplatin-resistant cells. The putative target gene ATXN3 was predicted by bioinformatic analysis and confirmed by dual-luciferase reporter assay. Importantly, ATXN3 was responsible for the regulatory effects of miR-619-5p on biological behaviors of cisplatin-resistant OSCC cells. Moreover, miR-619-5p mimics and ATXN3-siRNA significantly enhanced ATXN3 knockdown in both HN6/CDDPR and CAL27/CDDPR cells and inhibited expression of PI3K and AKT. *In vivo* evidences demonstrated that intratumoral injection of miR-619-5p agomir remarkably slowed down the growth of OSCC in xenograft mice. Collectively, microRNA-619-5p was the vital regulator for regulating cisplatin resistance of OSCC, which may be served as a potential therapeutic target.

## Introduction

Oral squamous cell carcinoma (OSCC) is the most frequent malignant tumor of the head and neck, covering nearly 90% of oral malignancy cases 1. OSCC is an epithelial malignant tumor with a strong metastatic ability 2. Risk factors of OSCC include smoking, drinking, betel-nut chewing, etc. 3, 4. Potential risk factors impair the ability of the human body to repair DNA damage caused by gene mutations and eventually result in immune deficiency 5. At present, surgical resection, chemotherapy and radiotherapy are mainly preferred to OSCC patients 6. Nevertheless, more than half of OSCC patients die of its cancer lesions or relevant complications within 5 years. The prognosis of OSCC is far from satisfactory 7, 8.

Chemotherapy is the major treatment for advanced or recurrent OSCC, which significantly prolongs survival of cancer patients and stimulates cancer regression 9, 10. Cisplatin is the well-recognized first-line chemotherapy drug that is commonly used in chemotherapy 11, 12. Nevertheless, about 70-80% recurrent OSCC patients are resistant to cisplatin and their clinical outcomes are largely restricted by drug resistance 13, 14. Cisplatin resistance, at present, is one of the biggest obstacles in the treatment of OSCC 15. Therefore, understanding molecular mechanisms of OSCC resistance and developing an effective method to reverse drug resistance are of great significance, aiming to improve clinical outcomes of OSCC 16.

MicroRNAs are small molecular, single-stranded, noncoding RNAs, with 22-29 transcripts in length 17. They are able to regulate gene translation and post-transcription 18. It is well known that microRNAs induce degradation of target mRNAs or inhibit protein translation *via* recognizing and binding their 3'-UTR, thereby participating in biological and cancerous processes 19. A growing number of evidences have shown that microRNAs are involved in the proliferation, apoptosis, migration, metastasis and other events of tumor cells 19, 20. In addition, microRNAs are closely related to drug resistance in cancer cases 21. It is reported that miR-194-5p involves in OSCC cell proliferation and cisplatin-resistance by regulating HIF1α 22. STAT3/miR-21 axis has been reported to be a candidate therapeutic target for OSCC chemoresistance 23. MiR-22 has recently been reported to promote tongue squamous cell carcinoma to cisplatin by activating PI3K/AKT signaling pathway 24. Our previous research detected differentially expressed microRNAs in exosomes released from erlotinib-resistant and erlotinib-sensitive cells 25. We demonstrated that miR-619-5p served an anti-cancer role in OSCC cell lines. An earlier study reported that miR-619-5p enhanced the sensitivity of pancreatic cancer to gemcitabine 26. MiR-619-5p shows a potential influence in chemotherapy resistance. Its specific role in drug sensitivity of OSCC cell lines, especially cisplatin resistance, however, remains unclear.

In the present study, we intended to explore the role of microRNA-619-5p in both *in vitro* and *in vivo* development of OSCC. Our results showed that overexpression of miR-619-5p could inhibit the activity of the PI3K/AKT signaling, and inhibit migratory and invasive functions of OSCC cells* in vitro*. Importantly, ectopically expressed microRNA-619-5p or deficiency of its target ATXN3 would enhance the sensitivity of OSCC cells to cisplatin. Our findings provide an experimental evidence that microRNA-619-5p was a potential target for cisplatin treatment combined with novel combination therapy of OSCC.

## Materials and methods

### Subjects and samples

A total of 40 primary OSCC cases treated in Stomatological Collage of Nanjing Medical University from 2017 to 2018 were retrospectively analyzed for their histological and clinical data. The information of recruited patients was listed in detail (Table 1). Patients did not receive any anti-cancer treatment prior to surgical resection. Non-metastatic primary OSCC tissues and adjacent non-tumoral ones were harvested during surgery and stored at -80 °C for use. Written informed consent was obtained before sample collection. This study got approval of the Ethic Committee of Nanjing Medical University.

### Cell culture

Six human cell lines (HOK, Leuk-1, HN4, HN6, CAL27 and UMSCC38) were obtained from ATCC. They were cultivated in DMEM + 10% FBS (CellMax cell Technology (beijing) Co.,Ltd SA311.02) in a humidified incubator at 37 °C, 5% CO_2_. For establishing cisplatin-resistant cell lines, HN6 and CAL27 cells were exposed to gradient concentration of cisplatin (CDDP) for over 6 months until they developed resistance to 2 μmol/ml and 1 μmol/ml cisplatin, respectively, that were, HN6/CDDPR and CAL27/CDDPR cells. They were cultivated in DMEM containing 2 μmol/ml and 1 μmol/ml cisplatin, respectively. Human embryonic kidney (HEK) 293T was purchased from Shanghai Institutes for Biological Sciences Cell Resource Center. The 293T cells were maintained in DMEM, high glucose supplemented with 10%FBS (CellMax cell Technology (beijing) Co., Ltd SA311.02).

### Cell transfection

MiR-619-5p mimics, miR-619-5p inhibitor, si-ATXN3, and negative control were obtained from GenePharma, Shanghai, China. Cells were seeded in a 6-well plate and transfected using Lipofectamine 2000 (Invitrogen, Carlsbad, CA, USA). Transfection efficacy was examined by performing qRT-PCR and Western blot. The sequences were as follows: miR-619-5p mimics: sense 5'- GCUGGGAUUACAGGCAUGAGCC -3' and antisense 5'- CUCAUGCCUGUAAUCCCAGCUU -3'; mimic control: sense 5′-UUCUCCGAACGUGUCACGUTT -3′ and antisense 5′-ACGUGACACGUUCGGAGAATT -3′; miR-619-5p inhibitors: 5'- GGCUCAUGCCUGUAAUCCCAGC -3'; inhibitor control: 5′- CAGUACUUUUGUGUAGUACAA -3′; si-ATXN3-1: 5'- GGACCUAUCAGGACAGAGUTT -3'; si-ATXN3-2: 5'- GGACAGAGUUCACAUCCAUTT -3; si-ATXN3-3: 5'- GCAAAAGCAGCAACAGCAGTT -3; si-NC: 5'- GGGTATCGACGATTACAAA -3'.

### Cell proliferation assay

Cell viability was assessed by CCK-8 assay. Transfected cells were seeded in a 96-well plate (1×10^3^ cells/well). Optical density at 450 nm was measured using the CCK-8 kit (Dojindo, Japan), followed by depicting cell viability curves.

Cell clonality was assessed by colony formation assay. Transfected cells were seeded in a 6-well plate (3×10^2^ cells/well), and incubated for 2 weeks. Visible colonies were fixed in formaldehyde, dyed in crystal violet and captured under a microscope (DM4000B, Leica, Germany). Colonies were calculated using ImageJ software.

### Cell invasion assay

Cell invasiveness was assessed by wound healing assay and Transwell assay. Transfected cells seeded in a 12-well plate were cultivated to 100% confluence. A scratch was made on the monolayer cells using a 200 µL pipette tip. Cells were washed in PBS and cultured in serum-free medium for 12 h. Wound healing images were captured at 0 and 12 h.

Transwell chambers (8 μm) pre-coated with diluted Matrigel (BD Biosciences) were prepared. Transfected cells were seeded in the chamber (1×10^5^ cells/chamber) and cultured for 24 h. Invasive cells were then fixed in paraformaldehyde, dyed in crystal violet and captured for three randomly selected views per chamber.

### qRT-PCR

Total RNA was extracted using TRIzol and reversely transcribed to cDNA using 5×PrimeScript RT Master Mix. qRT-PCR was conducted on the ABI 7900 qRT-PCR system (Applied Biosystems, Grand Island, NY) following protocols of SYBR Premix Ex Taq kit (Vazyme Biotech, Nanjing, China). GAPDH was the internal reference. Relative level was calculated by 2^-ΔΔCT^ method. The primers were listed as follows: GAPDH: F: 5'- GAAGGTGAAGGTCGGAGTC -3', R: 5'-GAGATGGTGATGGGATTTC -3'; ATXN3: F: 5'- GTTGGCTCCAGACAAATAAACATGG -3', R: 5'- AAGTGAGCCTTCTTGTTTCTCGT -3'; PIK3R1: F: 5'- CAGCAACCTGGCAGAATTACGA -3', R: 5'- TGACAGGATTTGGTAAGTCCAGGAG -3'; AKT1: F: 5'- AATACCTGGTGTCGGTCTCA -3', R: 5'- TCGAGCTCATCCTAATGGAG -3'.

### Western blot

Total protein was lysed for 30 min in the lysate buffer (Beyotime, Shanghai, China) and washed in pre-cold PBS. The protein was separated by SDS-PAGE, and loaded on PVDF membrane. Blockage of non-specific antigens was conducted in 5% skim milk for 2 h. Membranes were immunoblotted with primary and secondary antibodies for the indicated time points. Bands were finally exposed using Immobilon Western Chemiluminescent HRP Substrate for quantifying the grey value using the ImageQuantLAS 4000 mini biomolecular imager. The following antibodies were used for Western blot analyses: E-cadherin (#3195, CST), N-cadherin (ab18203, Abcam), Snail (#3879, CST), Slug (#9585, CST), Vimentin (#5741, CST), β-actin (AP0733, Bioworld, China), ATXN3 (#13505-1-AP, Proteintech), PI3K (#ab86714, abcam), AKT (#4691, CST), p-AKT (#66444-1-lg, Proteintech).

### Dual-luciferase reporter assay

MiRNA response elements of microRNA-619-5p (wild-type and mutant-type) in ATXN3 3'-UTR were amplified by qRT-PCR, and cloned into pmirGLO vector separately. Luciferase vectors were co-transfected into cells with microRNA-619-5p mimics, microRNA-619-5p inhibitor or negative control for 48 h, respectively. Later, luciferase intensities of Firefly and Renilla were measured to calculate the relative luciferase activity of each sample. Each experiment was repeated in triplicate.

### Bioinformatics

The target genes of miR-619-5p were predicted by three coputer-aided algorithms 27-29, namely TargetScan Release 7.0 (http://www.targetscan.org/vert_71/), miRTarBase (http://mirtarbase.mbc.nctu.edu.tw/) 28 and miRanda (http://www.microrna.org/microrna/home.do). The target genes were selected only when they were positive in all three algorithms. The ATXN3 expression data for OSCC was processed and analyzed by GEPIA, a web server for cancer and normal gene expression profiling and interactive analyses 30.

### Immunofluorescence staining

5×10^4^ cells were implanted on a slide and cultivated for 12 h, fixed in 2% methanol for 30 min and washed in PBS for three times. Later, cells were induced with PBS containing 0.5% Triton X-100 for 20 min, and incubated with primary antibodies at 4 °C overnight. After washing in PBS for three times, cells were induced with FITC or Cy3-labeled anti-rabbit IgG (1:200, Proteintech) for 1 h, followed by DAPI labeling in the dark. Immunofluorescence images were captured under a fluorescence microscope (DM4000B, Leica, Germany) and analyzed by ImageJ software.

### Immunohistochemistry

Paraffin-embedded tissues collected from nude mice were subjected to antigen retrieval in EDTA buffer (pH=9) for 15-min heating. After cooling, endogenous peroxide in tissues was blocked by incubating in 3% H_2_O_2_, which were washed in PBS twice and blocked in 5% skim milk for 1 h. Tissues were immunoblotted with primary and secondary antibodies for indicated time points, followed by counter-staining with hematoxylin, dehydration and fixation. Immunoreactive score (IRS) was assessed by the product of staining intensity and staining degree (0 = no obvious staining, 1 = weak staining, 2 = moderate staining, 3 = strong staining and 4 = extremely strong staining). The score of positive-stained cell ratio was categorized into 0-1: 0-25%, 1-2: 26-50%, 2-3: 51-75% and 3-4: 76-100%. Total score of immunohistochemistry was yielded by the product of IRS and score of positive-stained cell ratio, ranging from 1-12 (≥4 indicated a high expression). The following antibodies were used for analyses: ATXN3 (#13505-1-AP, Proteintech), Ki67 (#27309-1-AP, Proteintech).

### TCGA data analysis

A profile containing ATXN3 expressions in OSCC and/or adjacent non-tumoral tissues from 564 OSCC patients was downloaded from TCGA, and data were analyzed by BRB-ArrayTools Ver 4.5.

### Animal experiments

Animal experiments were approved by the Committee on Animal Ethics and Use, Nanjing Medical University, and all procedures were in accordance to laboratory animal welfare. Five-week-old male nude mice were obtained from Animal Core Facility of Nanjing Medical University, and habituated in SPF environment with 12h/12h light cycle. 1×10^7^ HN6 or HN6/CDDPR cells suspended in Matrigel were administrated into the back of each mouse. 5 nmol AgomicroRNA-619-5p or negative control diluted in 50 µL of PBS was administrated in nude mice with an interval of three days, for 6 times totally. Tumor width and length were measured at a fixed time point every three days. After 18 days, mice were anesthetized for sacrifice, and their tumor tissues were collected for experiments. Tumor volume (mm^3^) = Tumor width (mm) ^2^ × tumor length (mm) / 2.

### Statistical analysis

The student's *t*-test and chi-square test were used for analyzing continuous and categorical variables, respectively. Data from three independent experiments were expressed as mean ± SD. GraphPad Prism 7.0 was used for statistical analysis. *P* < 0.05 considered as statistically significant (*P<0.05, **P<0.01, ***P<0.001).

## Results

### MiR-619-5p is down-regulated in OSCC

**Figure 1:**
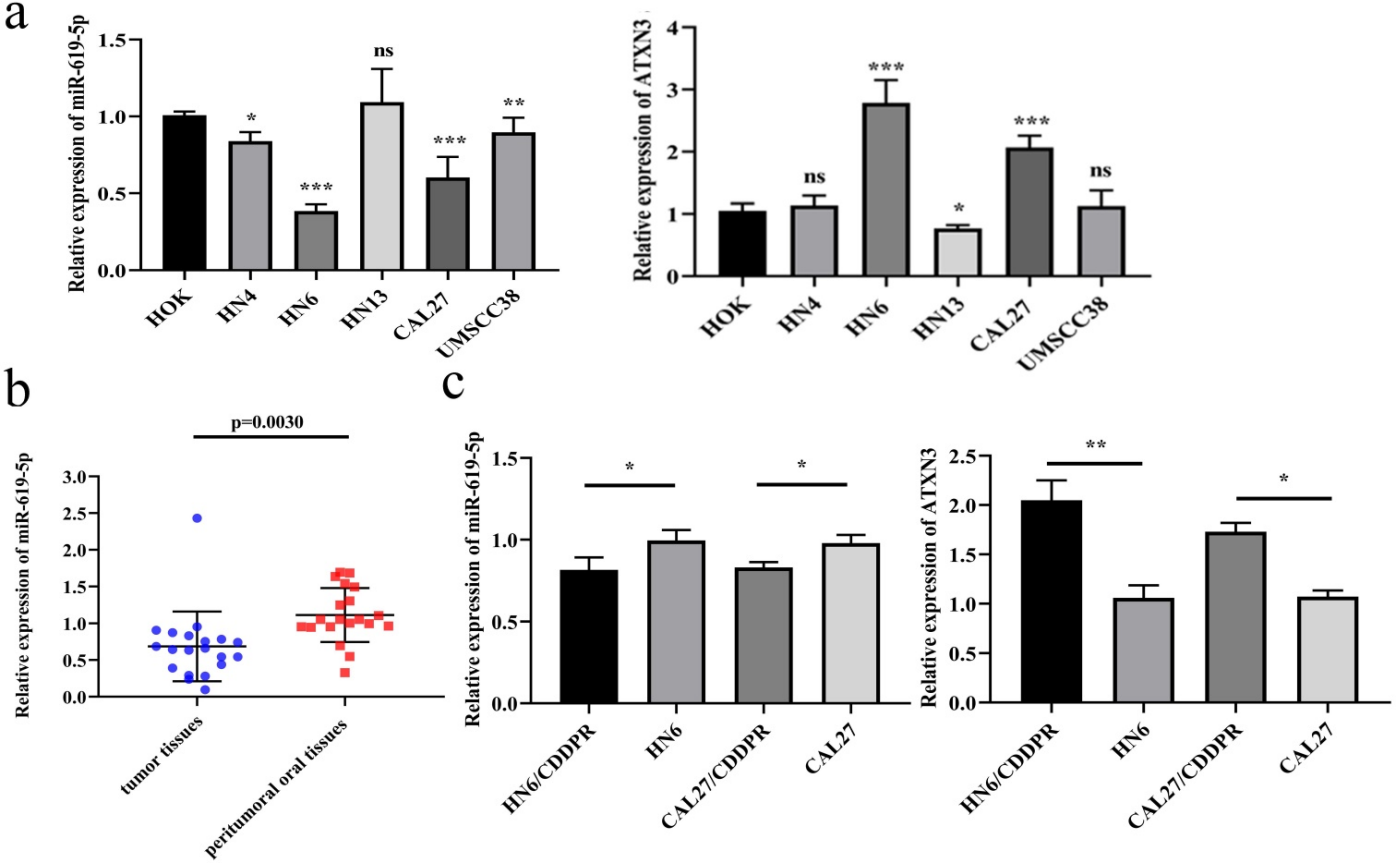
** Expression of miR-619-5p in OSCC**. (a) Relative miR-619-5p and ATXN3 expression in OSCC cell lines. (b) Patients' tumor tissues and peritumoral oral tissues (n=20) were collected. Expression of miR-619-5p in tumor tissues and the corresponding peritumoral tissues were detected by qRT-PCR analysis. (c) Real-time RT-PCR assay was performed to detect expression of miR-619-5p and ATXN3 in OSCC cells and cisplatin resistant cells. Data were shown as mean ± S.D. The symbols *, ** and *** represented great significant difference (*p* < 0.05, *p* < 0.01 and* p* < 0.001) by two-tailed Student's t-test.

To identify the biological function of miR-619-5p in OSCC, its level was first examined in OSCC cell lines. Compared with the oral epithelial keratinocytes HOK, miR-619-5p was down-regulated in OSCC cell lines, and the expression of ATXN3 was moderately increased in OSCC cell lines (Figure 1a). Meanwhile, miR-619-5p was down-regulated in OSCC tissues than that of paracancerous ones (Figure 1b). In addition, we compared differential level of miR-619-5p and ATXN3 in CDDP-resistant OSCC cell lines (HN6/CDDPR and CAL27/CDDPR) and their parental cells, and miR-619-5p level was markedly lower in CDDP-resistant cells compared with that of parental ones. Conversely, elevated expression level of ATXN3 was detected in CDDP-resistant cells (Figure 1c). The above data suggested that miR-619-5p was down-regulated in OSCC cells and tissues, which could be involved in OSCC development.

### MiR-619-5p inhibits proliferation and arrests cell cycle progression of OSCC cells

**Figure 2:**
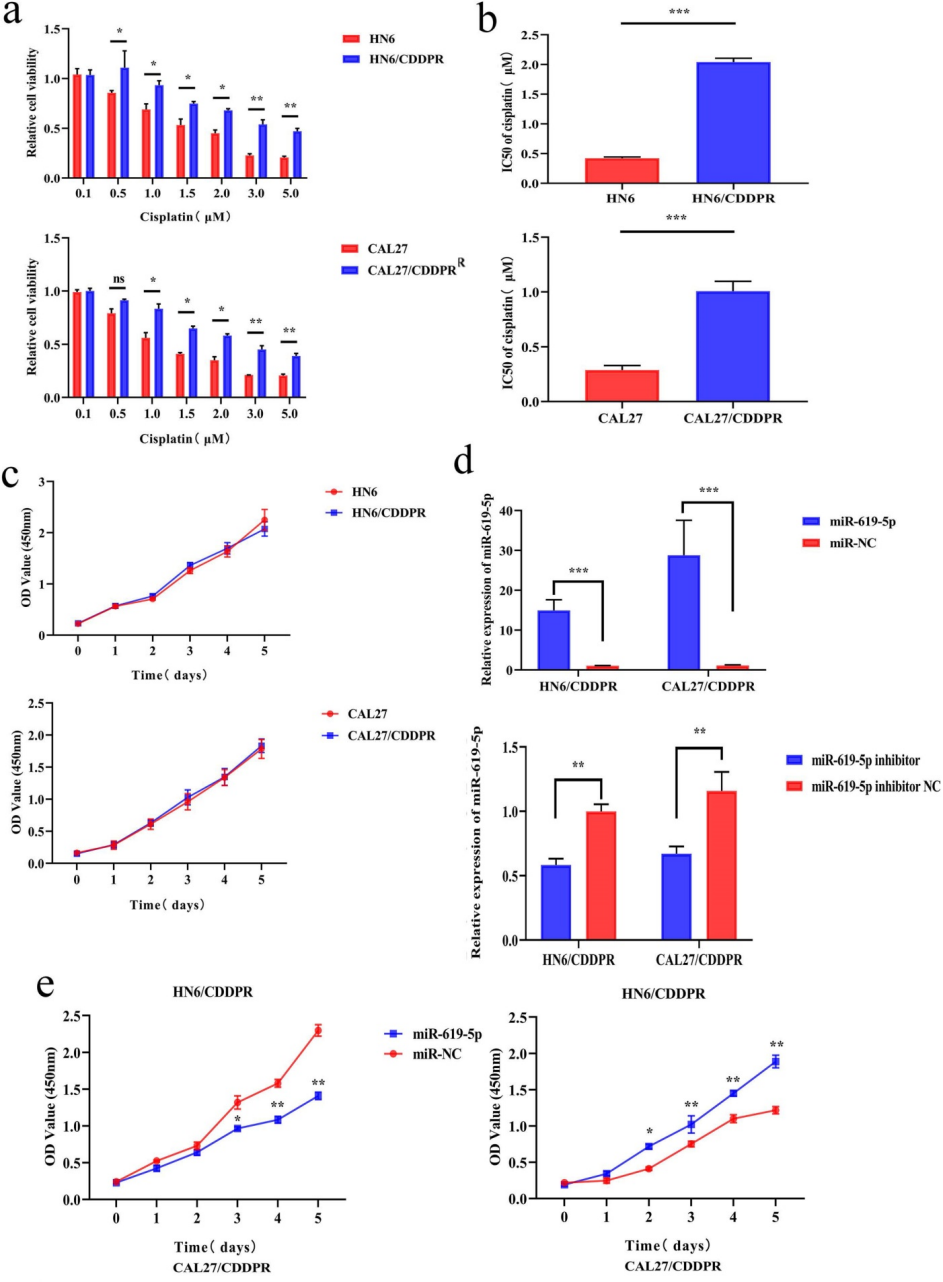

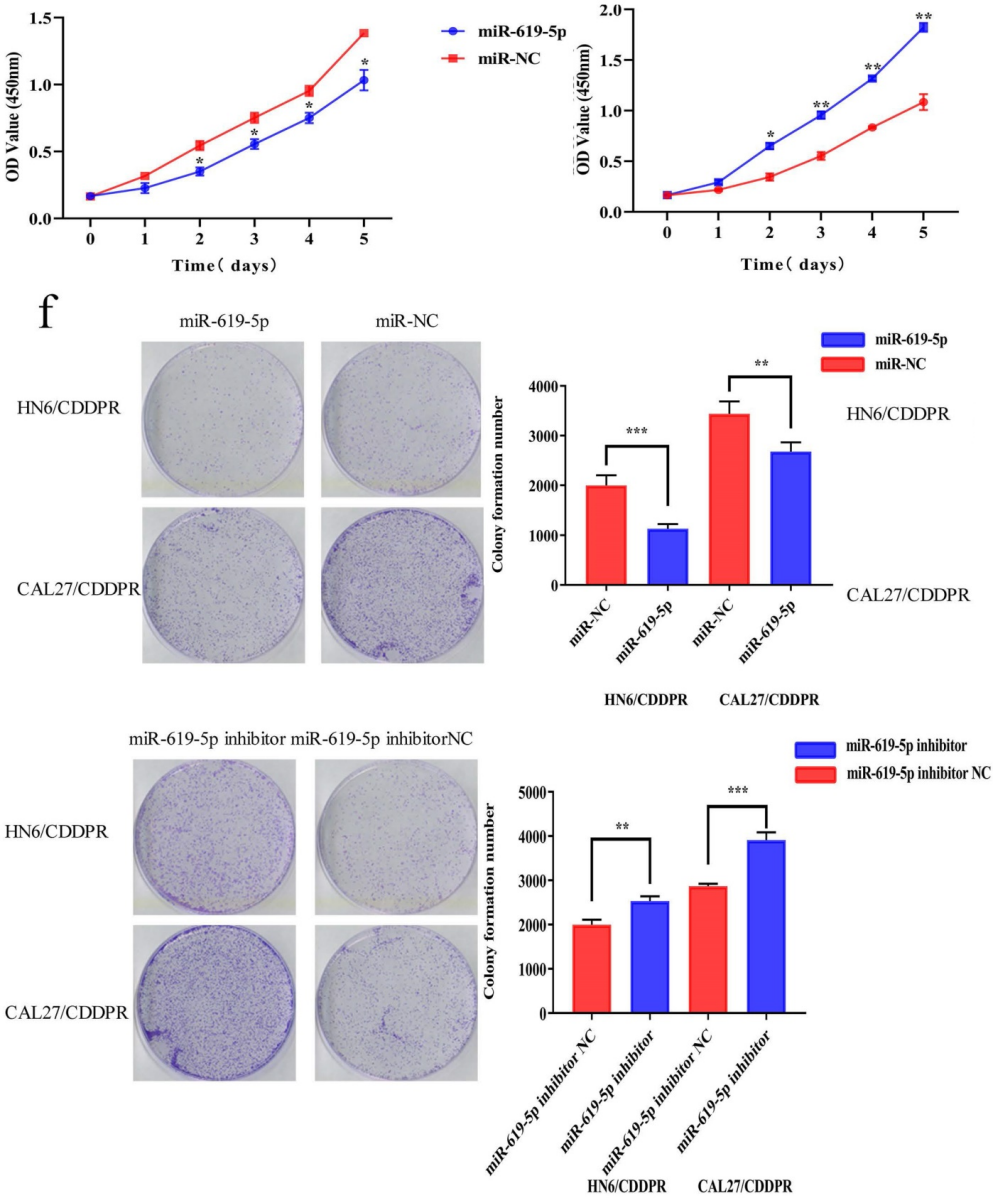
** Effect of miR-619-5p on cell proliferation and cell-cycle arrest *in vitro***. (a) CCK8 assays were performed to evaluate the cytotoxicity of different concentrations of cisplatin to HN6, HN6/CDDPR, CAL27 and CAL27/CDDPR. (b) IC50 of cisplatin to OSCC cell lines. (c) Comparison of proliferation of OSCC cell lines and OSCC cisplatin-resistant cell lines. (d) qRT-PCR detected the expression of miR-619-5p following transfection with miR-619-5p mimics and inhibitor in HN6 and CAL27 OSCC cells. (e and f) HN6/CDDPR and CAL27/CDDPR cells were transfected with miR-619-5p mimics or inhibitor, and proliferation ability of cells was detected by CCK8 assay and colony formation assay. *p < 0.05, **p < 0.01, ***p < 0.001.

To study the resistance of cisplatin on OSCC, we continuously exposed the OSCC cell lines HN6 and CAL27 to cisplatin. The obtained cells were named as HN6/CDDPR and CAL27/CDDPR cells. Chemoresistance of cisplatin-resistant OSCC cell lines to cisplatin exhibited significant lower sensitivity to various concentrations of cisplatin compared to parental cell lines (Figure 2a), and we calculated that IC50 of cisplatin to HN6/CDDPR and CAL27/CDDPR (Figure 2b). Meanwhile, light microscopy studies revealed that HN6/CDDPR and CAL27/CDDPR didn't display a striking change in cell shape compared to parental cell lines. We next wanted to investigate the proliferative capacity of HN6/CDDPR and CAL27/CDDPR compared with parental cell lines. No significant difference in cell proliferation was found between parental cell lines and cisplatin-resistant cell lines (Figure 2c). Transfection efficacy of miR-619-5p mimics and inhibitor in OSCC resistant cells was tested (Figure 2d). Regulatory effect of miR-619-5p on proliferative ability of OSCC cells was examined by CCK-8 and colony formation assay. It is shown that overexpression of miR-619-5p attenuated proliferative ability of HN6/CDDPR and CAL27/CDDPR cells, whilst knockdown of miR-619-5p obtained an opposite result (Figure 2e,f).

### MiR-619-5p inhibits migration and invasion of OSCC cells

**Figure 3:**
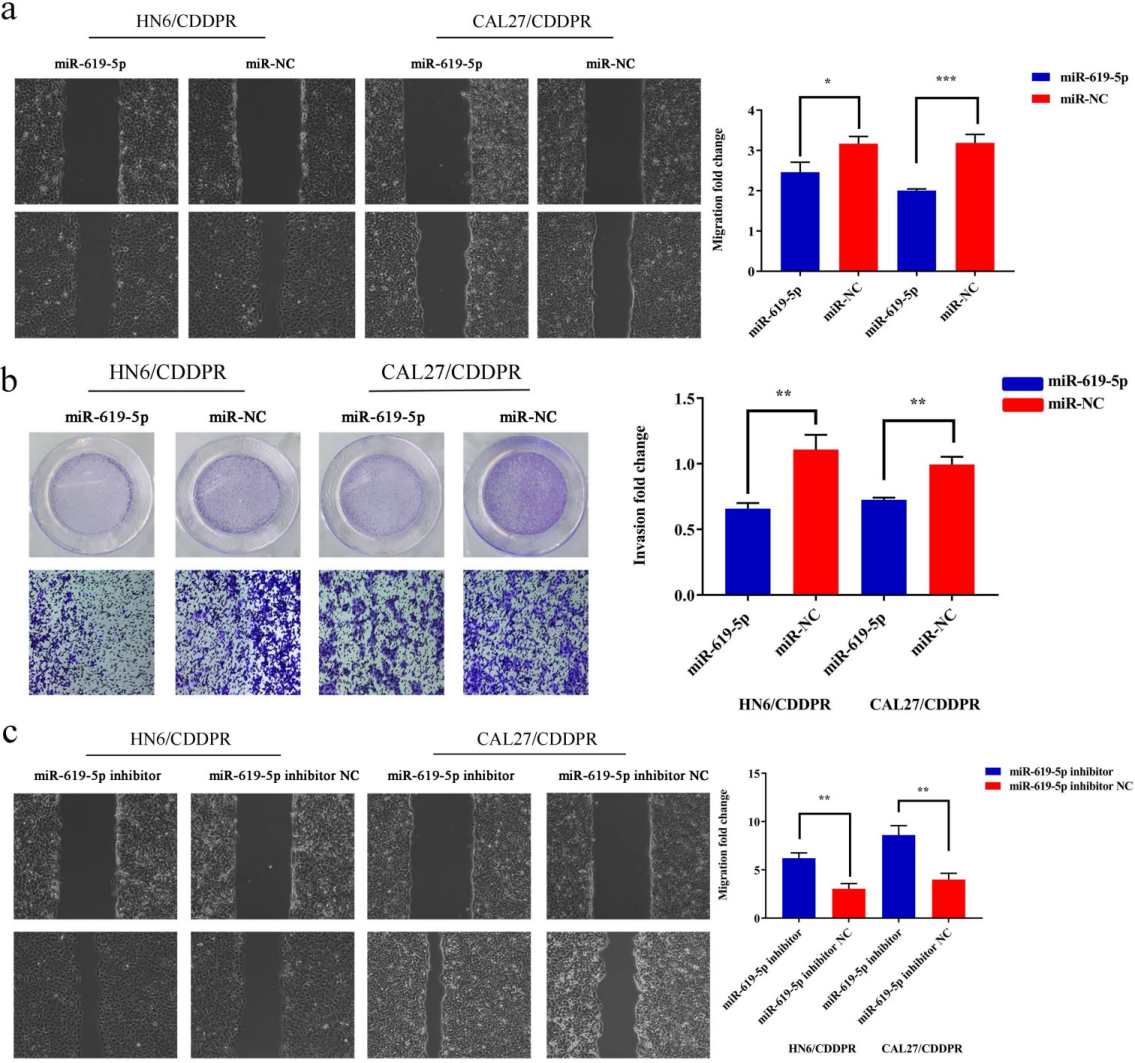

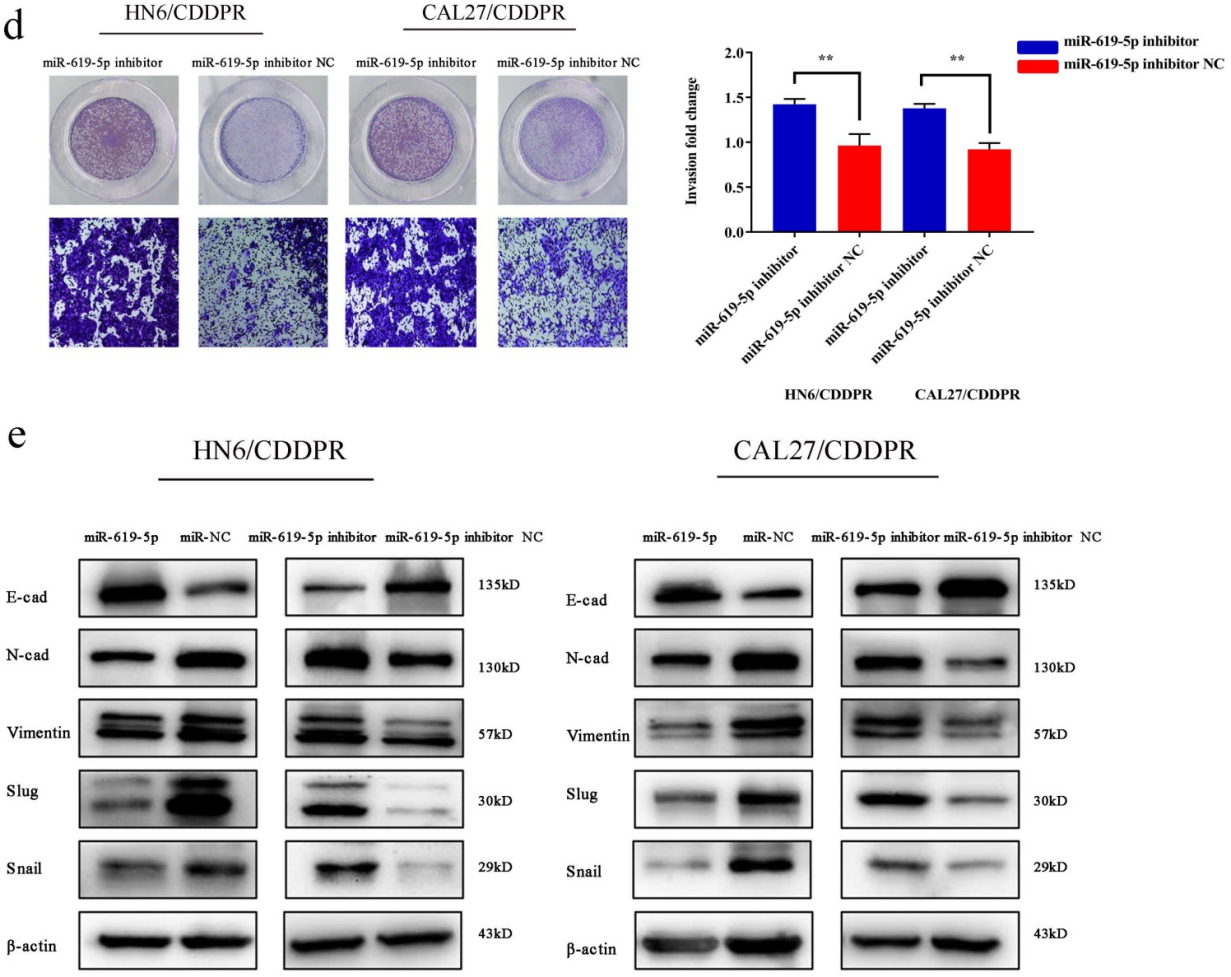

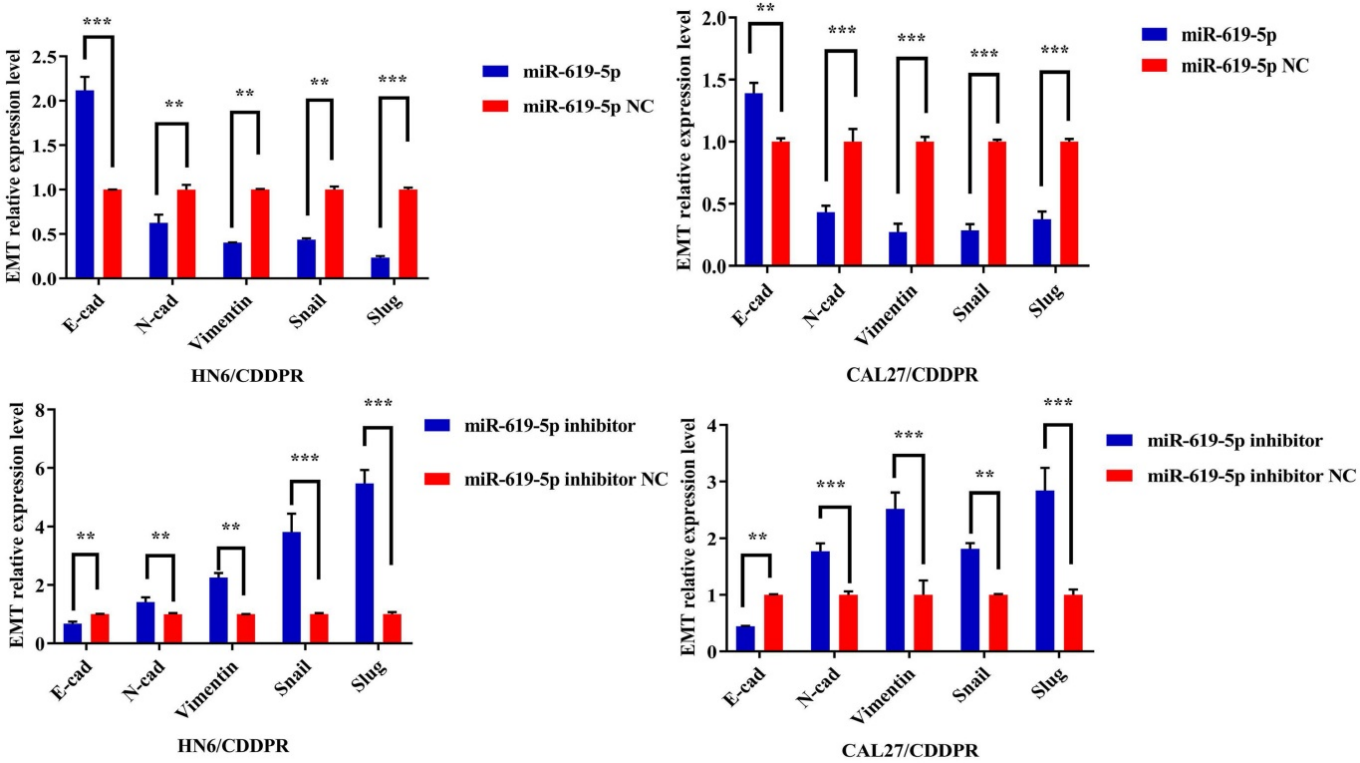
** Effect of miR-619-5p on cell migratory and invasive activity in OSCC cisplatin resistant cells**. (a) Wound healing assay showed that overexpression of miR-619-5p mimics decreased the cell migration ability. Representative image depicting the beginning (t=0h) and the end (t=12h) of recording were shown. (b) Transwell assays of HN6/CDDPR and CAL27/CDDPR cells with stable miR-619-5p mimics. Magnification: × 100. (c and d) The opposite results were observed on miR-619-5p inhibitor overexpression by Wound healing and Transwell assays. (e) MiR-619-5p overexpression restrained EMT process in HN6/CDDPR and CAL27/CDDPR cells and miR-619-5p knockdown promoted EMT process.

Migratory and invasive abilities of OSCC cells mediated by miR-619-5p were assessed by wound healing assay and Transwell assay. As shown in Figure 3a and 3b, overexpression of miR-619-5p suppressed migratory and invasive abilities of HN6/CDDPR and CAL27/CDDPR cells. However, knockdown of miR-619-5p accelerated their metastatic ability (Figure 3c, d). Accumulating evidences have proven that EMT is closely related to tumor infiltration and metastasis 31, 32. EMT is frequently observed during OSCC development 33. Generally, EMT is featured by down-regulation of epithelial markers (E-cadherin) and up-regulation of mesenchymal markers (N-cadherin and vimentin) 6. Here, we analyzed the role of miR-619-5p in mediating expression levels of EMT-associated markers. It is shown that overexpression of miR-619-5p blocked EMT in OSCC cells, while knockdown of miR-619-5p accelerated it (Figure 3e).

### ATXN3 is predicted to be the target gene of miR-619-5p

**Figure 4:**
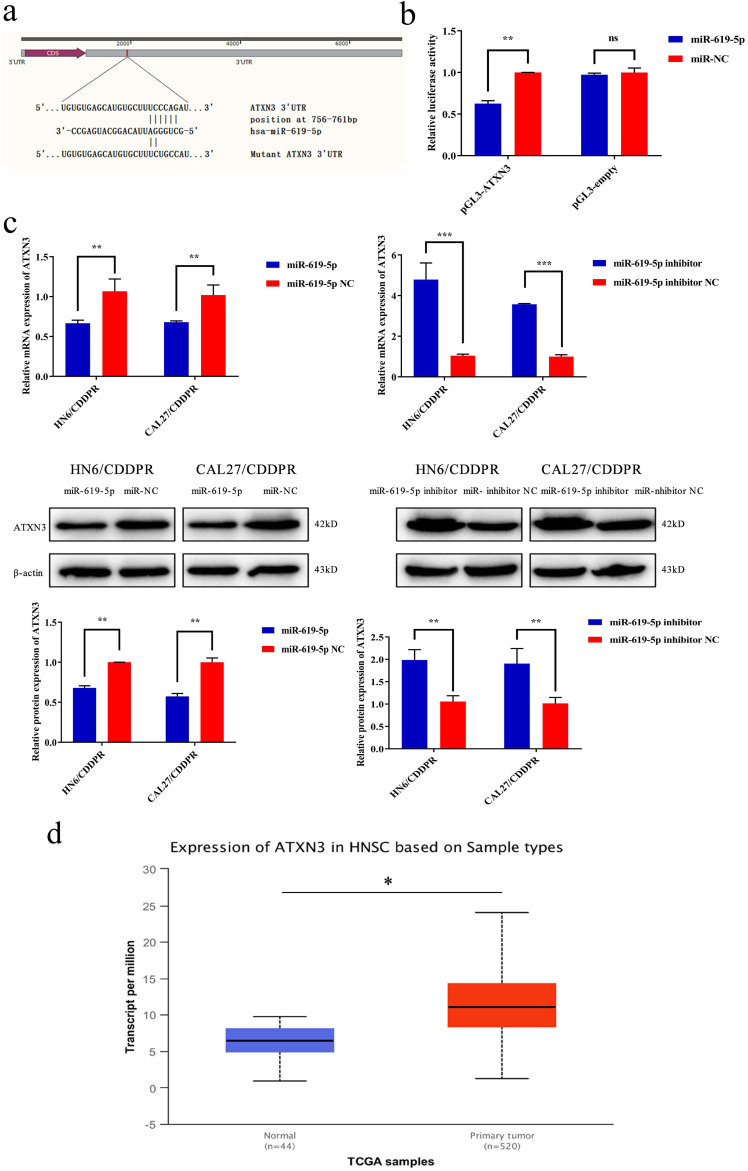
** ATXN3 was associated with miR-619-5p expression**. (a) Sequences of wild-type and mutant target sites for miR-619-5p in ATXN3 were shown. (b) Verification of ATXN3 as a target gene of miR-619-5p by the dual luciferase reporter assay. (c) Real-time RT-PCR assay and western blotting analyses were performed to detect the expression of ATXN3 at the mRNA and protein levels, in CDDPR cells treated with miR-619-5p mimics and inhibitor, respectively. (d) Expression of ATXN3 mRNAs in primary tumor and normal samples in the TCGA HNSCC database.

It is well known that microRNAs inhibit translation of target genes by binding their 3'-UTR through the partial homologous sequences. To predict potential targets of miR-619-5p, we searched publicly available algorithms, including TargetScanmiRTarBase and miRanda. And many candidate target genes for miR-619-5p were predicted on public miRNA databases and the mRNA expression levels of these genes were measured at 24 h after transfection miR-619-5p or miR-NC (Supplemental Figure 1). Of these, we found that ATXN3 was significantly suppressed and was identified to be the target of miR-619-5p, which was further verified. The homologous sequence in 3'-UTR of miR-619-5p and ATXN3 was shown in Figure 4a. 293T cells were used in this experimental due to their high transfection efficiency. Wild-type and mutant-type ATXN3 luciferase vectors (wt-ATXN3 3'-UTR and mut-ATXN3 3'-UTR) were transfected into 293T cells with either miR-619-5p mimics or negative control. Notably, luciferase activity was significantly inhibited by co-transfection of miR-619-5p mimics and wt-ATXN3 3'-UTR, while co-transfection of miR-619-5p mimics and mut-ATXN3 3'-UTR did not result in a significant change of luciferase activity (Figure 4b). Hence, it is confirmed that ATXN3 was the target of miR-619-5p. Furthermore, regulatory effect of miR-619-5p on ATXN3 level in both HN6/CDDPR and CAL27/CDDPR cells was examined. Overexpression of miR-619-5p down-regulated both mRNA and protein level of ATXN3, while knockdown of miR-619-5p up-regulated their levels (Figure 4c). We thereafter analyzed RNA-seq of OSCC patients in TCGA. Interestingly, ATXN3 was up-regulated in OSCC tissues than that of adjacent non-tumoral ones (Figure 4d). It is concluded that ATXN3 was the direct target of miR-619-5p, which exerted a carcinogenic role in OSCC development.

### ATXN3 is responsible for cisplatin resistance of OSCC cells mediated by miR-619-5p

**Figure 5:**
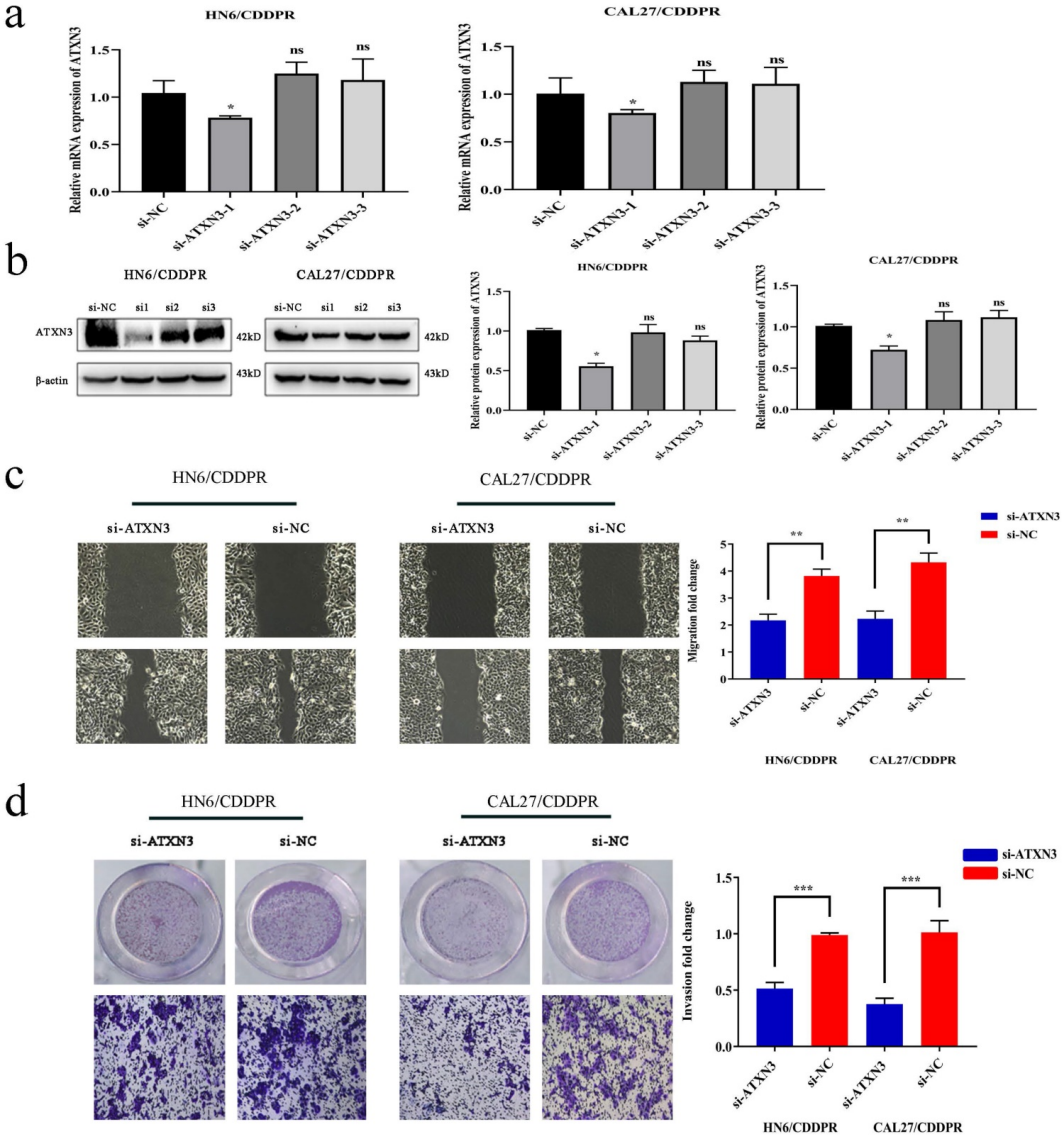
** Downregulation of ATXN3 inhibited cell migratory, invasive activity and cell-cycle in both CDDPR OSCC cells**. (a) Real-time RT-PCR and (b) western blotting analyses were performed to measure the expression of ATXN3 in HN6/CDDPR and CAL27/CDDPR cells after ATXN3 siRNA transfection. (c) Ability of HN6/CDDPR and CAL27/CDDPR cells to migrate after scratch wound injury were inhibited by transfected si-ATXN3. (d) Transwell assays were performed to identify the capacity of cell invasion after transfection with si-ATXN3.

To clarify the involvement of ATXN3 in cisplatin resistance of OSCC cells, HN6/CDDPR and CAL27/CDDPR cells were transfected with ATXN3 siRNAs. It is shown that transfection of ATXN3 siRNA1 effectively down-regulated ATXN3, which was used in the following experiments (Figure 5a, b). Transfection of si-ATXN3 significantly inhibited migration and invasion in HN6/CDDPR and CAL27/CDDPR cells (Figure 5c, d).

### Synergistical role of miR-619-5p and ATXN3 in regulating OSCC cells

**Figure 6:**
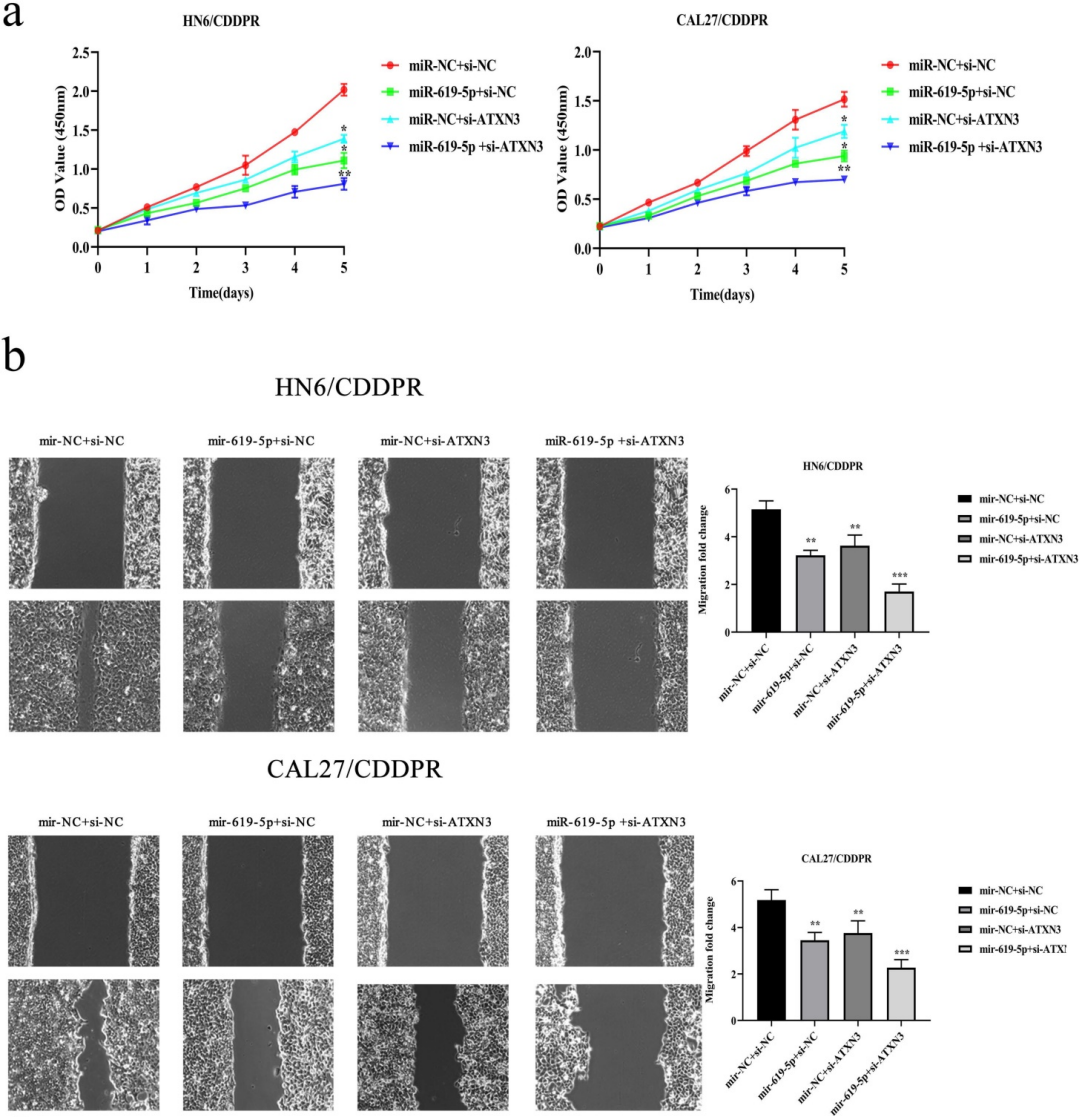

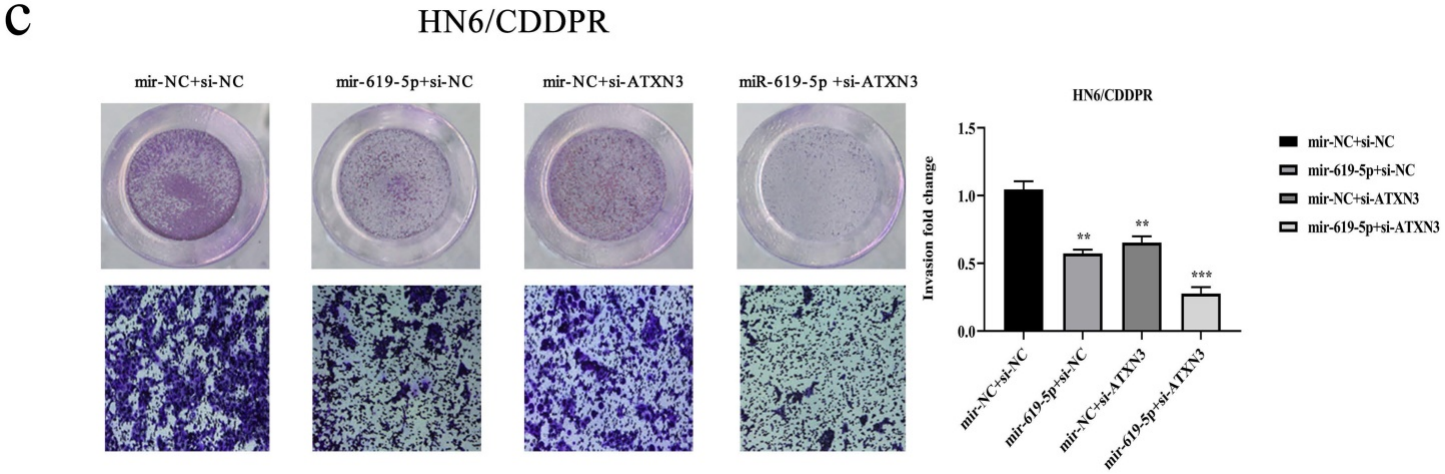

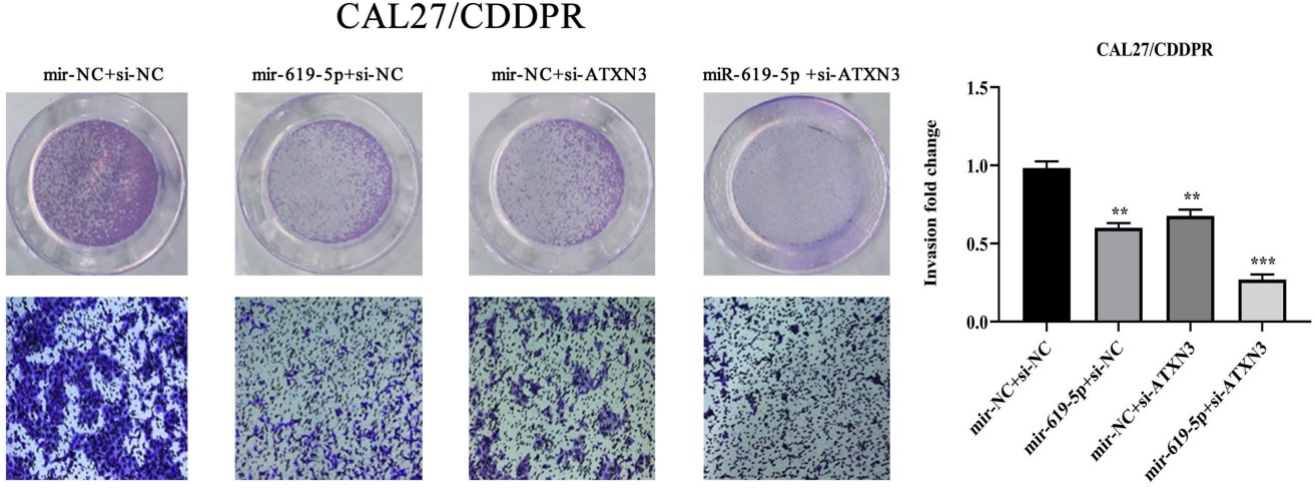

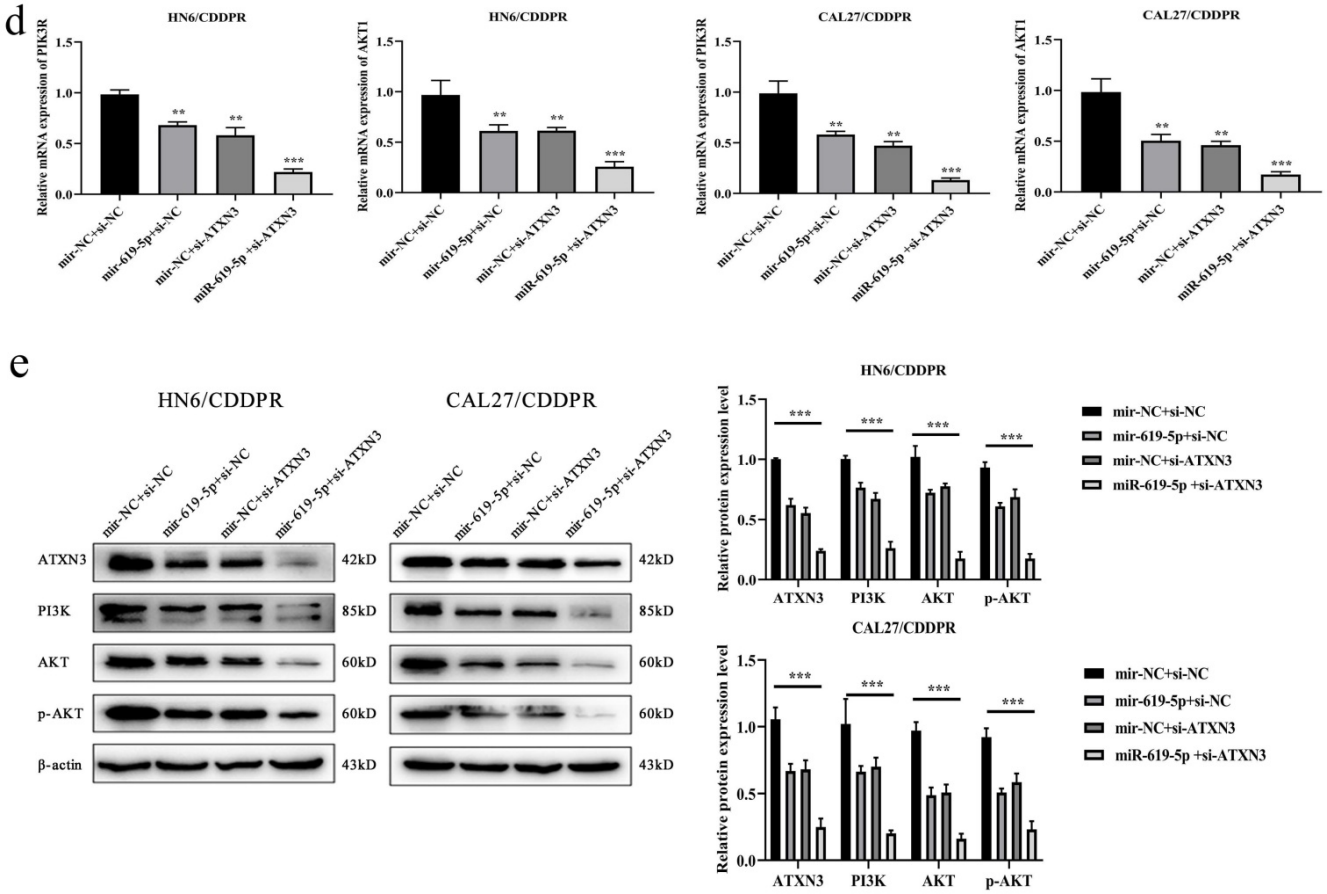
** Combination of miR-619-5p and si-ATXN3 treatment showed enhanced ATXN3 inhibition *in vitro***. (a) HN6/CDDPR and CAL27/CDDPR were transfection with miR-619-5p and si-ATXN3 alone and combination of them, and the cell viability was detected by CCK8 assay. (b and c) Representative images showed that the effect of different combination treatments on HN6/CDDPR and CAL27/CDDPR migration and invasion. (d) Real-time RT-PCR and (e) western blotting analyses were performed to measure the expression of ATXN3, PI3K and AKT after treatment with si-ATXN3 or a combination of both.

A combination of miRNA and siRNA is an attractive method to enhance the targeted efficacy on anti-cancer treatment. Here, we analyzed the synergistic role of overexpressed miR-619-5p and silenced ATXN3 in cell behaviors of OSCC. To explore the effects of miR-619-5p and si-ATXN3 on cell viability, CCK-8 assay was carried out after transfection with miR-619-5p and si-ATXN3 alone and combination of them (Figure 6a). However, in HOK, only the combined use slightly inhibited cell viability (Supplemental Figure 2). These data provided more evidence that miR-siRNA combination therapy worked in drug-resistant cells. We found that transfection of miR-619-5p or si-ATXN3 alone reduced cell proliferation ability, and the combined transfection enhanced this effect. As shown in Figure 6b and 6c, HN6/CDDPR and CAL27/CDDPR cells were co-transfected with miR-619-5p mimics and si-ATXN3. Compared with controls, a single transfection of either of them could attenuate migratory and invasive abilities of HN6/CDDPR and CAL27/CDDPR cells, and the inhibitory effects were more strengthened by their co-transfection. It is suggested that a combination of miR-619-5p overexpression and si-ATXN3 exerted a pronounced synergistic role in regulating migration and invasion of OSCC cells. Considering that miR-619-5p was screened from erlotinib-resistant cells, and EGFR triggered phosphatidylinositol 3 kinase (PI3K)/AKT pathway 34, 35. Meanwhile, previous studies have shown that the PI3K and AKT signaling are all involved in cancer metastasis and drug resistance 36. Here, we detected mRNA and protein levels of PI3K and AKT in OSCC cells mediated by miR-619-5p and ATXN3. Overexpression of miR-619-5p or knockdown of ATXN3 could down-regulate their levels, and the down-regulated trends were more obvious by co-transfection of miR-619-5p mimics and si-ATXN3 (Figure 6d,e). The above data highlighted the vital function of miR-619-5p in OSCC development, as well as the synergistic role of ATXN3.

### MiR-619-5p regulates cisplatin resistance of OSCC *in vivo*

**Figure 7:**
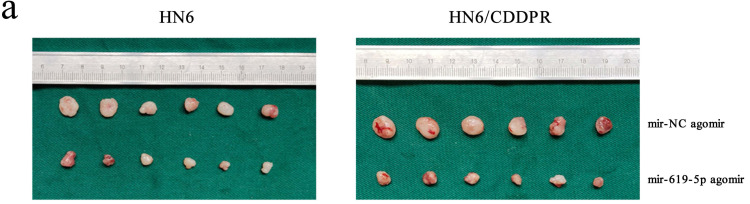

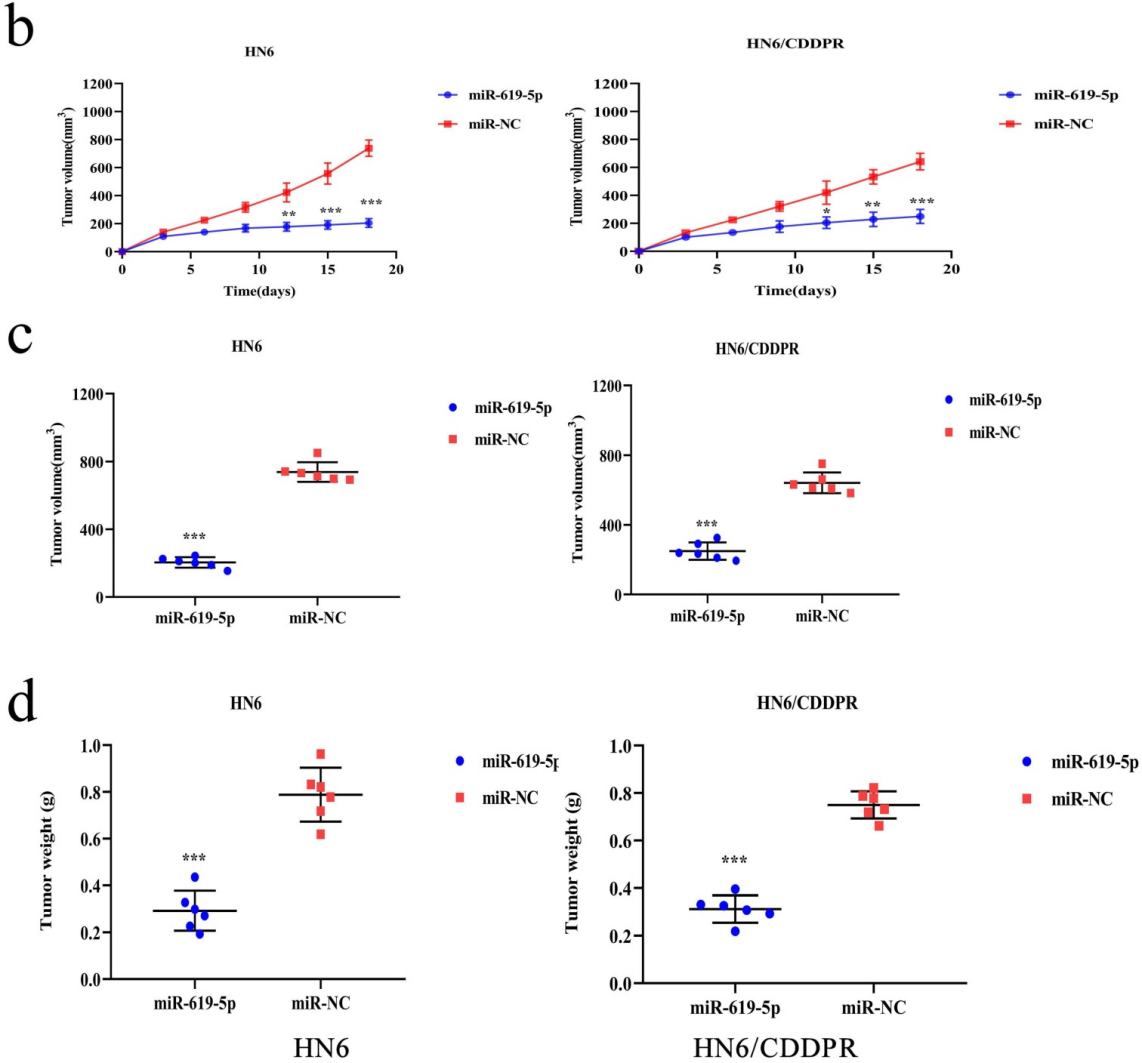

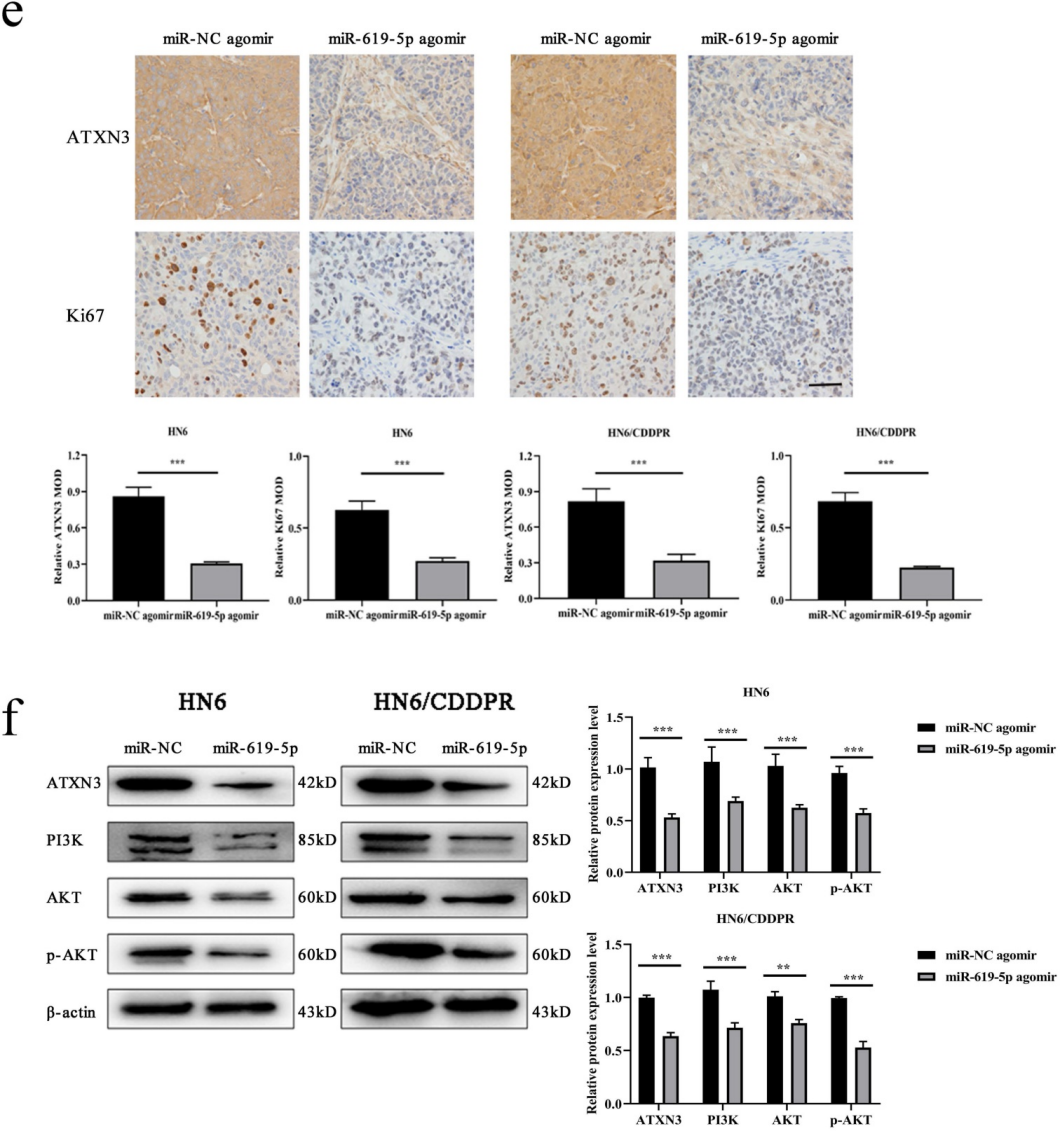
** Effect of miR-619-5p on tumor growth in xenograft model and in cisplatin resistant xenograft model**. (a) MiR-619-5p agomir inhibited tumor growth *in vivo*. Images showed representative tumors that dissected from our model. (b, c and d) Growth curves of xenograft tumors (b), tumor volumes (c) and tumor weight (d). (e) Representative H&E staining of tumor tissues and IHC staining of ATXN3 and Ki67. Scale bar, 50 µm. (f) Western blotting analysis was performed to detect the expression of ATXN3, PI3K, AKT and p-AKT *in vivo*. *p < 0.05, **p < 0.01, ***p < 0.001.

As shown in Figure 7a (left panel), the tumor size in the miR-619-5p agomir group was much smaller than those in the NC agomir group. Consistently, the mean volume and weight of the tumors extracted from the miR-619-5p agomir group were lower than that of the NC agomir group (Figure 7b, c and d, left panel). To validate the regulatory effect of miR-619-5p on cisplatin sensitivity of OSCC *in vivo*, a xenograft model in cisplatin-resistant OSCC-bearing mice was established by implanting HN6/CDDPR cells. OSCC-bearing mice with an intratumoral injection of miR-619-5p agomir had smaller cancer tissues than those with an injection of NC agomir (Figure 7a, right panel), which was also reflected by the growth curve of OSCC (Figure 7b, right panel). As expected, the average volume and weight of OSCC harvested from OSCC-bearing mice with an intratumoral injection of miR-619-5p agomir were lower than those with an injection of NC agomir (Figure 7c and d, right panel). In addition, immunostaining of Ki67 and ATXN3 in collected OSCC tissues was conducted. In miR-619-5p agomir group, positive expression of ATXN3 was significantly reduced (Figure 7e). At last, the anti-cancer mechanism of miR-619-5p in OSCC *in vivo* was explored. Similar to *in vitro* results we yielded, miR-619-5p was able to down-regulate *in vivo* protein levels of PI3K and AKT in OSCC-bearing mice as well (Figure 7f).

## Discussion

Chemotherapy has been extensively applied for cancer treatment, including OSCC 37, 38. Surgery combined with postoperative chemotherapy is the first-line therapeutic strategy for OSCC 39. The aim of chemotherapy is to stimulate cancer regression after surgical resection and prevent relapse 38. During the past decades, cancer treatment has been rapidly progressed. Chemotherapy resistance, however, is still the huge obstacle in clinical treatment of OSCC 40. In recent years, the role of microRNAs in altering chemotherapy resistance has emerged, which can be utilized for reversing cancer resistance. Its particular mechanism is required to be fully analyzed 41.

Our previous results have found differentially expressed microRNAs in exosomes released from erlotinib-resistant and erlotinib-sensitive cells. It is identified that miR-619-5p exerts an oncogenic role in OSCC cell lines 25. A relevant study reported that miR-619-5p suppresses proliferative and invasive capacities of colorectal carcinoma cells 42. A latest report demonstrated that miR-619-5p enhances sensitivity of pancreatic cancer cells to gemcitabine *via* activating the Wnt/β-catenin signaling pathway 26. It is concluded that miR-619-5p not only serves as a tumor biomarker, but also a potential therapeutic target for preventing tumor growth and drug resistance.

In the present study, we found that miR-619-5p was down-regulated in cisplatin-resistant OSCC cells than that of parental cells. In addition, miR-619-5p is reported to participate in the proliferation, migration and invasion of cancer cells. Identifying target genes regulated by a certain miRNA is of great significance to highlight its biological functions. Here, we predicted the target gene of miR-619-5p using bioinformatic methods, which was further verified by dual-luciferase reporter assay. Our results demonstrated that miR-619-5p accelerated cisplatin resistance in OSCC by downregulating ATXN3 both *in vitro* and *in vivo*.

Human ataxin-3 gene locates on chromosome 14q21 that encodes ATXN3, which contains (CAG)n repeats in the coding region 43. The specific sequence in ATXN3 encodes polyQ-expanded ataxin-3 44. Its abnormal amplification leads to Machado-Joseph disease (MJD), also known as spinocerebellar ataxia type 3 (SCA3), an autosomal dominant neuropathy 45. The expansion of (CAG)n repeats from the normal 12-44 to 52-86 is one cause of MJD 46. Meanwhile, ATXN3 is a type of deubiquitinases that maintains cell homeostasis and links to carcinogenesis. ATXN3 is highly expressed in testicular cancer that promotes cancer cell proliferation. It is also involved in breast cancer metastasis. Through deubiquitinating transcription of KLF4, ATXN3 is responsible for inducing lung cancer development 47. Our results showed that knockdown of ATXN3 suppressed migratory and invasive abilities in cisplatin-resistant OSCC cells. Notably, a negative correlation was detected between microRNA-619-5p and ATXN3 levels in OSCC cells and tissues. A combination of miRNA-based and siRNA-based treatment provides a dual-inhibitory effect on target proteins. In this paper, co-transfection of miR-619-5p mimics and si-ATXN3 in OSCC cells significantly strengthened the downregulation of ATXN3. At the meantime, combination treatment by miR-619-5p mimics and si-ATXN3 presented a better inhibitory effect on migratory and invasive abilities of cisplatin-resistant OSCC cells in comparison to single treatment, which was a novel strategy for miRNA-based and siRNA-based treatment of OSCC.

Our previous study screened out miR-619-5p from exosomes of erlotinib-resistant cells 25. Erlotinib is a classic inhibitor of the EGFR signaling pathway, and PI3K/AKT is a critical downstream signaling of EGFR, which is also essential in developing acquired drug resistance 48. A relevant study implied that miR-32-5p in exosomes is able to induce multidrug resistance of liver cancer cells through activating the PI3K/AKT signaling pathway 49. Here, transfection of either miR-619-5p mimics or si-ATXN3 could inhibit the activity of the PI3K/AKT signaling pathway, and the inhibitory outcome was much more pronounced by co-transfection of them.

Collectively, we proved the role of miR-619-5p in inhibiting both *in vitro* and* in vivo* migration and invasion of OSCC. Through directing targeting ATXN3, miR-619-5p inactivated the PI3K/AKT signaling pathway to induce cell cycle blockage and inhibition of migration and invasion of HN6/CDDPR and CAL27/CDDPR cells. In the OSCC xenograft model, miR-619-5p level was negatively correlated to that of ATXN3, and both of them were related to cisplatin sensitivity in OSCC. Our findings provide an experimental evidence that miR-619-5p/ATXN3 axis may be a vital target for the diagnosis and treatment of cisplatin resistance in OSCC.

## Supplementary Material

Supplementary figures.

## Figures and Tables

**Table 1:** Clinical features of 40 patients with OSCC

No.	Age	Sex	Location	T	N	M	Differentiation
1	59	F	Gingiva	1	0	0	Well
2	69	M	Buccal	2	0	0	Well
3	64	M	Buccal	2	0	0	Well
4	74	M	Palate	2	0	0	Poor
5	57	M	Gingiva	3	0	0	Moderate
6	67	F	Buccal	2	0	0	Well
7	54	F	Oropharynx	2	0	0	Poor
8	72	M	Palate	2	0	0	Well
9	64	M	Gingiva	4a	0	0	Poor
10	65	F	Buccal	2	0	0	Moderate
11	38	M	Tongue	2	0	0	Moderate
12	60	F	Tongue	4a	0	0	Poor
13	67	F	Tongue	4a	0	0	Moderate
14	79	M	Palate	4a	0	0	Poor
15	49	M	Tongue	3	0	0	Poor
16	43	M	Buccal	4a	0	0	Moderate
17	61	M	Gingiva	2	0	0	Poor
18	54	M	Buccal	1	0	0	Moderate to well
19	81	M	Gingiva	2	0	0	Moderate
20	68	F	Buccal	1	0	0	Moderate
21	50	M	Gingiva	2	1	0	Moderate
22	67	M	Buccal	2	1	0	Moderate
23	63	M	Tongue	3	1	0	Poor
24	64	M	Gingiva	4	2a	0	Poor
25	65	F	Tongue	2	1	0	Poor
26	38	F	Buccal	1	2b	0	Moderate
27	60	M	Tongue	2	1	0	Well
28	67	M	Tongue	3	2c	0	Well
29	79	F	Buccal	4a	2b	0	Well
30	56	M	Gingiva	4a	2b	0	Poor
31	78	M	Buccal	3	2b	0	Well
32	69	M	Gingiva	2	2b	0	Poor
33	68	F	Buccal	3	1	0	Moderate
34	74	F	Buccal	4a	2a	0	Poor
35	57	M	Tongue	4a	1	0	Poor
36	67	M	Gingiva	4a	1	0	Poor
37	54	F	Gingiva	4a	1	0	Poor
38	72	M	Gingiva	3	1	0	Moderate
39	74	F	Gingiva	2	1	0	Poor
40	71	F	Tongue	3	1	0	Moderate

OSCC oral squamous cell carcinoma, F female, M male; TNM classification and tumor stage were determined by the Union for International Cancer Control (UICC); OSCC without metastatic primary tumors (No. 1-20); OSCC with associated regional nodal metastases (No. 21-40).

## Abbreviations

OSCC: oral squamous cell carcinoma
ATXN3: ataxin3
CDDPR: cisplatin-resistance
