# Involvement of miR-619-5p in resistance to cisplatin by regulating ATXN3 in oral squamous cell carcinoma: Erratum

**DOI:** 10.7150/ijbs.94832

**Published:** 2024-02-25

**Authors:** An Song, Yuanyuan Wu, Weiming Chu, Xueming Yang, Zaiou Zhu, Enshi Yan, Wei Zhang, Junbo Zhou, Xu Ding, Jie Liu, Hongxia Zhu, Jinhai Ye, Yunong Wu, Yang Zheng, Xiaomeng Song

**Affiliations:** 1 Jiangsu Key Laboratory of Oral Diseases, Nanjing Medical University, Nanjing, Jiangsu, People's Republic of China.; 2 Department of Oral and Maxillofacial Surgery, Affiliated Hospital of Stomatology, Nanjing Medical University, Nanjing, Jiangsu, People's Republic of China.; 3 Clinical Medical College, Yangzhou University, Yangzhou, Jiangsu, People's Republic of China.; 4 Department of Stomatology, the Affiliated People's Hospital of Jiangsu University, Zhenjiang 21200, Jiangsu Province, China.; 5 Department of Anesthesiology, Affiliated Hospital of Stomatology, Nanjing Medical University, Nanjing, Jiangsu, People's Republic of China.; 6 Department of Stomatology, Nanjing Integrated Traditional Chinese and Western Medicine Hospital, Nanjing, Jiangsu, People's Republic of China.

In our paper, the author noticed an error in Figure 6b. We checked the original data again and made sure that the conclusion of the article was not affected by the error. In this regard, all authors have agreed to the erratum, and we apologize for any inconvenience caused by the negligence in our work.

Figure 6b should be corrected as follows.

## Figures and Tables

**Figure 6b:**
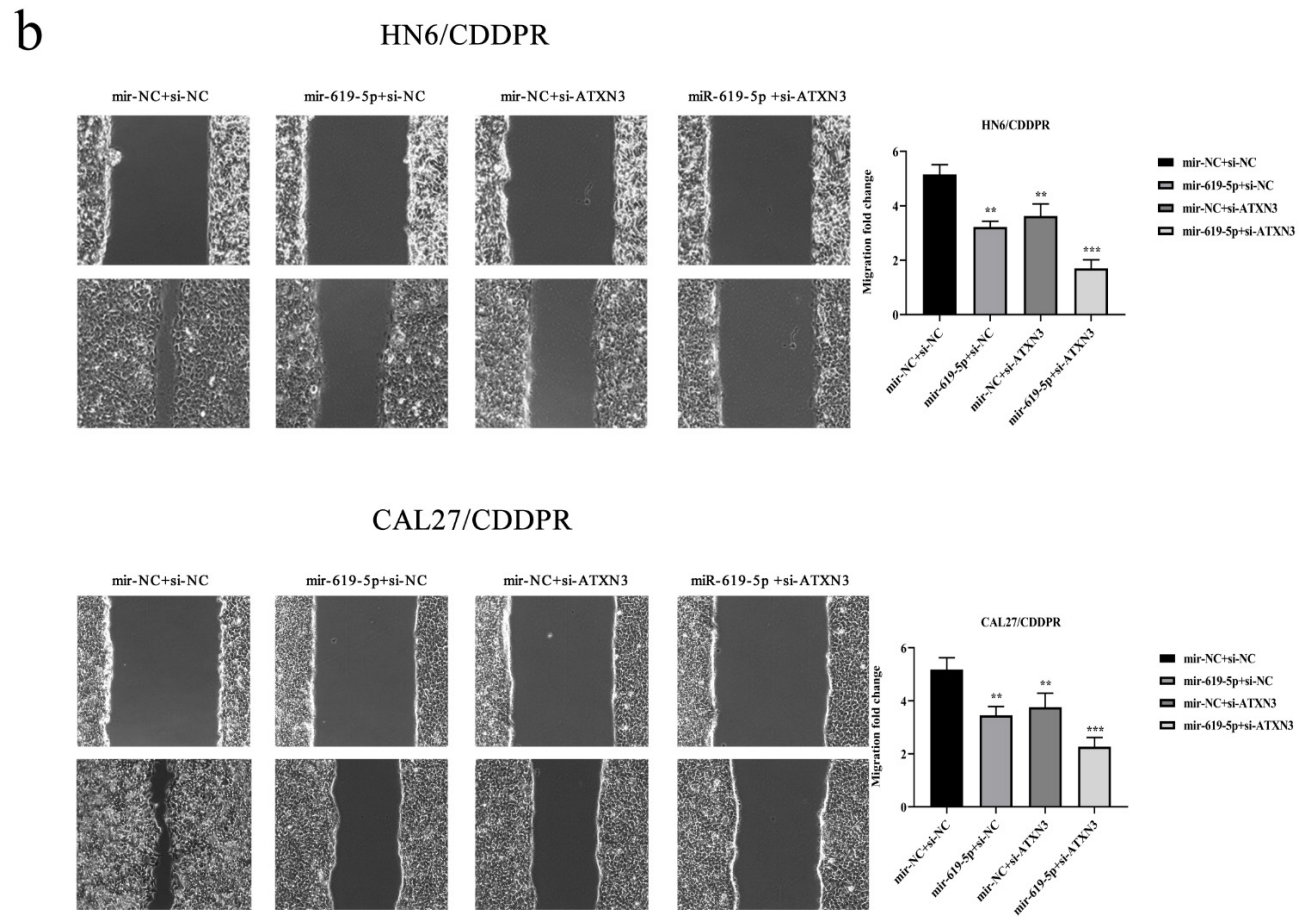
Correct image.

