# Supplementary material for: Involvement of miR-619-5p in resistance to cisplatin by regulating ATXN3 in oral squamous cell carcinoma
Source: Int J Biol Sci. 2021 Jan 1;17(2):430–47. doi: 10.7150/ijbs.54014 (PMC7893581; doi:10.7150/ijbs.54014)
Supplement: Supplementary file 1 — Supplementary figures. [file ijbsv17p0430s1.pdf]

Supplemental Figure 1 Validation of the predicted miR-619-5p target genes.

Supplemental Figure 2 HOK was transfection with miR-619-5p and si-ATXN3 alone and combination of them, and the cell viability was detected by CCK8 assay.

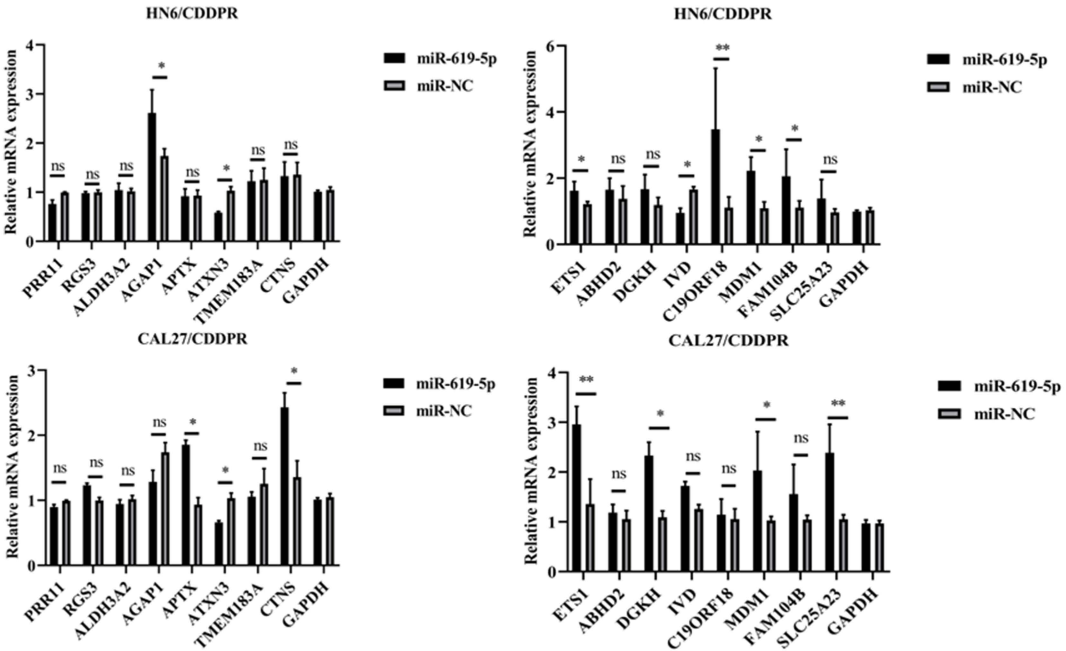

HN6/CDDPR
Relative mRNA expression
miR-619-5p
miR-NC
PRR11
RGS3
ALDH3A2
AGAP1
APTX
ATXN3
TMEM183A
CTNS
GAPDH
HN6/CDDPR
ETS1
ABHD2
DGKH
IVD
C19ORF18
MDM1
FAM104B
SLC25A23
GAPDH
CAL27/CDDPR
CAL27/CDDPR

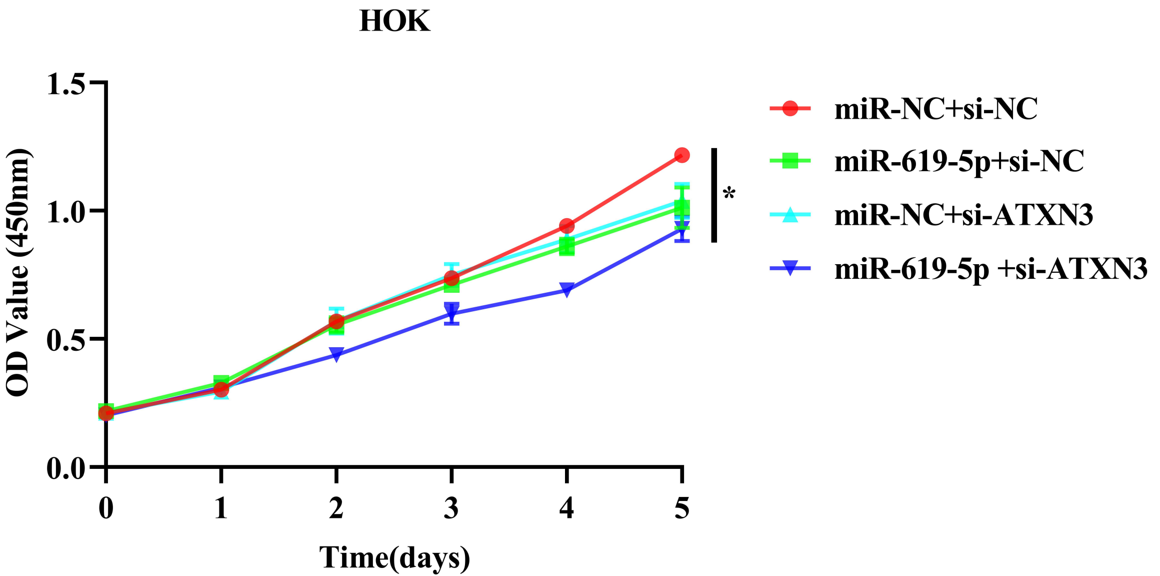
HOK
OD Value (450nm)
1.5
1.0
0.5
0.0
0
1
2
3
4
5
Time(days)
*
miR-NC+si-NC
miR-619-5p+si-NC
miR-NC+si-ATXN3
miR-619-5p +si-ATXN3
